# Efficacy and Tolerability of STOPAIN for a Migraine Attack

**DOI:** 10.3389/fneur.2015.00011

**Published:** 2015-02-04

**Authors:** Andrea St. Cyr, Ashley Chen, Kathleen C. Bradley, Hsiangkuo Yuan, Stephen D. Silberstein, William B. Young

**Affiliations:** ^1^Jefferson Headache Center, Department of Neurology, Thomas Jefferson University, Philadelphia, PA, USA

**Keywords:** acute migraine, acute migraine treatment, topical treatment, headache, migraine

## Abstract

**Objective:** To determine whether topical menthol 6% gel will relieve a migraine attack.

**Materials and Methods:** A single-center, open-label pilot trial of 25 patients with at least 1 year of diagnosed episodic migraine and <15 headache days per month. Patients treated one migraine attack with STOPAIN topical menthol 6% gel to skull base within 2 h of headache onset. Headache pain severity was assessed prior to and after gel application.

**Results:** Thirty-two patients enrolled and 25 completed the study. Prior to treatment, 7 patients had mild pain, 13 moderate pain, and 5 severe pain. Two hours following gel application, 7 (28%) patients had no pain, 7 (28%) mild pain, 6 (25%) moderate pain, and 5 (20%) severe pain. The majority of patients had similar pain intensity (8; 32%) or improvement (13; 52%). At 24-h, only two non-rescued patients still had mild headache. Of the 25 completers, 2 patients took rescue medication prior to the 2-h period, and an additional 10 patients rescued between 2 and 24 h.

**Conclusion:** Study results showed a significant improvement in headache intensity by 2 h after gel application. This pilot study shows STOPAIN gel may be effective in treating an acute migraine attack.

## Introduction

Migraine is a common headache disorder with an estimated 1-year prevalence of 36 million people (12% of the general population) in the United States (1), where estimated annual costs total as much as 17 billion dollars, including the direct costs of medications; office, clinic, or emergency department visits; laboratory and diagnostic services; and management of treatment side effects. Indirect costs from lost productivity in the workplace add substantially to the total (2). According to the World Health Organization, migraine is 19th cause of disability worldwide. Headache disorders impose burdens that include substantial personal suffering, impaired quality of life, and financial cost. Repeated headache attacks, and often fear of future attacks, damage family life, social life, and employment (3).

To date, formulations such as oral pill/powder, nasal spray, rectal suppository, and injectable medication are available options for acute migraine treatment. Topical treatments, in contrast, are interesting alternatives due to their accessibility, low cost, rapid onset of action, lack of systemic side effects, and the fact that they bypass the gastrointestinal tract.

Menthol is a natural compound manufactured in many topical medications. It has a number of potential peripheral and central analgesic effects. When administered systemically, it blocks voltage-gated calcium and sodium channels in superficial dorsal horn neurons, resulting in decreased neuronal excitability and blocked spontaneous synaptic transmission (4). Topical menthol blocks voltage-gated sodium channels in dorsal root ganglion neurons (5) and desensitizes nociceptive C-fibers; the sensitization of which is associated with cutaneous allodynia (6). The cooling sensation of menthol has been attributed to activation of transient receptor potential melastatin eight ion channels (7), which may be responsible for the analgesic effect (8). Thus, the molecular mechanism of menthol analgesia includes both central and peripheral effects. Anecdotal evidence suggests that they are effective for headache relief. Our study examined headache pain severity after applying 6% menthol gel (STOPAIN) topically.

## Materials and Methods

### Study design

We conducted a single-center, open-label pilot study that was approved by Thomas Jefferson University Institutional Review Board. Patients were recruited from outpatients who attended the Jefferson Headache Center between June and August of 2012. The study population consisted of 32 patients who had been diagnosed for 1 year with episodic migraine with or without aura according to the International Classification of Headache Disorders (2nd Edition-2004). Eligible patients between 18 and 65 years of age at screening were required to have 1–10 migraine attacks and no more than 15 total headache days per month. Subjects who had a diagnosis of basilar or hemiplegic migraine were excluded. Additional exclusion criteria included medical or psychiatric conditions that would increase the risk of adverse events or interfere with study assessments, participation in an investigational drug trial in the 30 days prior to the screening visit, and pregnancy or lactation. Women of childbearing potential were required to use effective contraception during participation.

Participants provided written informed consent prior to undergoing screening procedures. The screening visit (Visit 1) included a review of the patient’s medical and headache history and current medications. Vital signs and a urine pregnancy test were required for women of childbearing potential. Subjects were asked to treat a single migraine attack within 8 weeks of Visit 1. They were instructed to apply two pumps of the treatment gel from the metered dosing bottle to the area below and abutting the base of the skull to the base of the neck as well as behind and between the ears within 2 h of the onset of a migraine attack. If they had no relief after 2 h of gel application, they were permitted to use rescue medication.

Subjects were provided with a take-home diary. Within 1 h of headache onset, they were instructed to record pain severity (mild, moderate, or severe) and associated symptoms before applying the study gel, and at multiple time points (30, 60, and 90 min and 2, 4, and 24 h) after administering the gel. They also recorded the presence or absence of photophobia, phonophobia, nausea, and vomiting. Adverse events and rescue medications, including the time of treatment and dose, were to be recorded.

After treating the migraine attack and completing the diary, subjects were asked to return to Jefferson Headache Center for a final visit (Visit 2) or to return the diary and other study supplies by mail (shipping materials were offered to each participant). During the final visit, the study drug and diary were collected and reviewed, and any changes in concomitant medications and/or medical conditions since the screening visit were recorded.

### Study treatment gel

Active ingredients: Mentholum (l-menthol), Belladonna 3× HPUS, Nux Vomica 6× HPUS, *Iris Versicolor* 6× HPUS, and *Sanguinaria canadensis* 6× HPUS. Inactive ingredients: water (USP), ethanol (USP), cambopol polymer, propylene diglycol, and triethanolamine.

### Outcome measures and statistical analysis

The primary objective is to observe the responses after topical gel application in order to determine whether larger, well-controlled trials are warranted. The primary endpoint is the headache intensity reduction 2 h after treatment. An exploratory endpoint is the headache intensity reduction at 24 h after treatment. For statistical analysis, ordinal conversion of pain levels were assigned as 0 = no pain, 1 = mild pain, 2 = moderate pain, and 3 = severe pain. The Wilcoxon sign-rank test was used to compare pre-treatment pain severity with pain severity 2 h after gel application, as well as to compare pre-treatment pain severity with pain severity 24 h after gel application.

## Results

**Figure 1:**
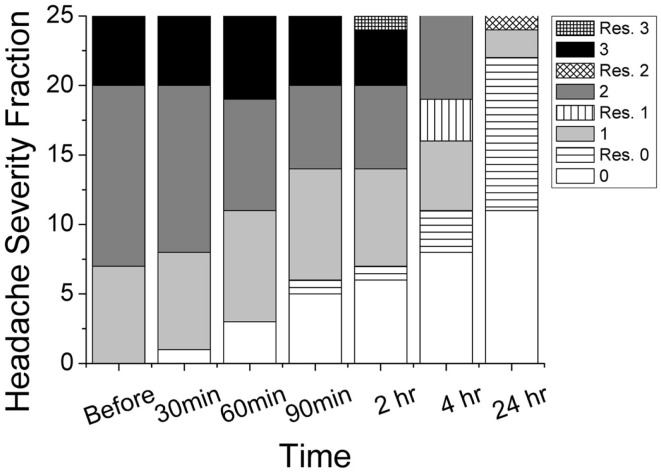
**Progression of reported headache intensity on four-point scale (0 = none, 1 = mild, 2 = moderate, 3 = severe) throughout the study period**. Rescued subjects (patterned) and non-rescued subjects (gray scale) were grouped separately. Total number of patients in this study was 25.

Thirty-two subjects with episodic migraine met the inclusion criteria and were enrolled in the pilot study. Of these, 25 subjects completed the study and seven were lost to follow up. Of the 25 completed subjects, 7 had mild pain, 13 had moderate pain, and 5 had severe pain before the topical treatment. Figure 1 illustrates the progression of headache intensity throughout the study period. Two hours after gel application, 7 subjects had no pain, 7 mild pain, 6 moderate pain, and 5 severe pain. Two completers rescued with medication due to progression from moderate to severe pain and were pain free afterwards. Twelve of the 23 non-rescue subjects (52%) reported improvement in headache pain by at least 1 severity level (e.g., from severe to moderate), 8 (35%) similar severity level, and 3 (13%) worse pain. Improvements were reported in 3 (43%), 7 (54%), 3 (60%) of mild, moderate, severe groups, respectively. After 24 h, 13 non-rescued subjects (11 pain free, 2 mild pain) showed pain improvement without any medication. Ten additional subjects reported the use of medication. The overall rates of rescue medication use are 3 (43%), 7 (46%), 3 (60%) for mild, moderate, severe groups, respectively. The total 12 rescued subjects were pain free except 1 having worsening moderate pain.

**Figure 2:**
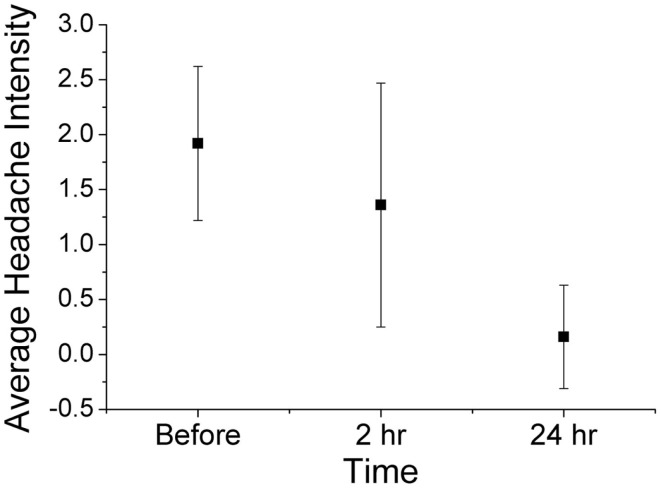
**Average (mean ± 1 SD) headache severity on four-point scale at before treatment, 2 h post treatment, and 24 h post treatment (all 25 completers)**. Wilcoxon sign-rank tests show significant reductions in pain intensity at both 2 h (*p* = 0.029) and 24 h (*p* < 0.001).

**Figure 3:**
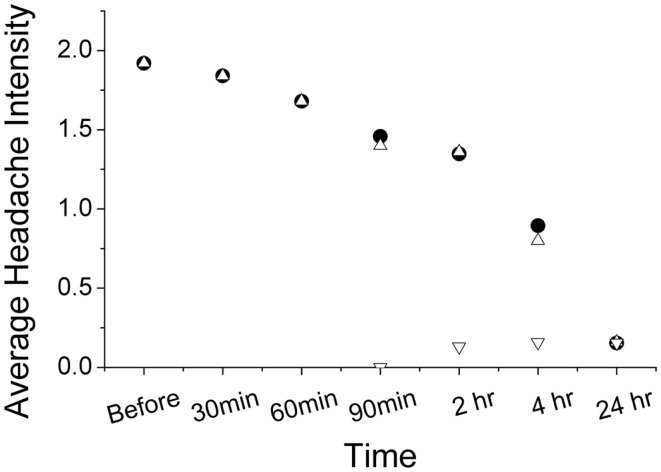
**Progression of average headache reported from subjects with (inverted open triangle) and without (open triangle) rescue medication use**. The pain progression of both non-rescued and overall subjects (solid circle) is nearly identical.

Using ordinal conversion of headache severity, the mean headache severity levels are at pre-treatment = 1.92 (close to moderate pain), at 2 h = 1.36 (between mild and moderate pain), and at 24 h = 0.16 (very mild pain) (Figure 2). Wilcoxon sign-rank test shows that a topical application of the study gel is significantly associated with a reduction in migraine severity after 2 h (*Z* = −2.18, *p* = 0.029) and after 24 h (*Z* = −4.342, *p* < 0.001). Figure 3 illustrates the overall progression of average pain intensity, as well as the rescued and non-rescued subjects, throughout the study period. Statistically, analysis on non-rescue subjects showed similar findings as overall subjects (data not shown).

Fifteen of the 25 subjects provided feedback regarding using one’s fingers to apply the gel. Twelve participants had no problems with the method of application and found the gel easy to use. Three participants said that they found the method acceptable, but complained that the gel could be messy, hard to pump, and difficult to apply if one had thick hair. Three participants said that the recommended method of application would not deter them from repeat use despite their complaints. One of the three participants with complaints said that a roll-on would be preferable to applying the gel with one’s fingers.

## Discussion

Given its unique properties, topical treatments for migraine headache may provide an alternative for episodic migraine sufferers. This open-label pilot study showed a statistically significant improvement in headache by at least one severity level in 52% of subjects 2 h after applying menthol 6% gel. Two-hour headache-free rate was 28%. Only 2 subjects (8%) required rescue medication before 2 h. This shows a potential pain-relieving property from topical menthol application. Interestingly, subjects with pre-treatment severe headache have higher rate of pain improvement after 2 h and also reported higher rate of rescue medication use within 24 h. Single application of menthol 6% gel is probably more useful for short-term ablation in migraine patients. After 24 h, most subjects, rescued or non-rescued, showed headache improvement, which could be attributed to the natural progression of the migraine.

Limitations of this study include the small sample size and open-label, non-placebo-controlled design. In previous studies conducted in 2010 by Borhani Haghighi et al., a 10% menthol solution applied to the forehead and temporal area showed significant improvement in headache pain and associated symptoms at 2 h compared to placebo in patients with episodic migraine without aura (9). Our findings of significant improvement after 6% menthol gel use provide a basis for further placebo-controlled studies of topical menthol treatment.

This open-label pilot study demonstrates that STOPAIN 6% menthol gel is safe and may be effective in the treatment of an acute migraine attack. Because of the limitations of this study, a larger placebo-controlled clinical trial is warranted.

## Conflict of Interest Statement

The authors declare that the research was conducted in the absence of any commercial or financial relationships that could be construed as a potential conflict of interest.
